# Preparation of ultrasound contrast agents: The exploration of the structure-echogenicity relationship of contrast agents based on neural network model

**DOI:** 10.3389/fonc.2022.964314

**Published:** 2022-10-05

**Authors:** Feng Li, Wensheng Xu, Yujin Feng, Wengang Wang, Hui Tian, Suhuan He, Liang Li, Bai Xiang, Yueheng Wang

**Affiliations:** ^1^ Department of Ultrasound, The Second Hospital of Hebei Medical University, Shijiazhuang, Hebei, China; ^2^ The First Outpatient Department of Hebei Province, Shijiazhuang, Hebei, China; ^3^ Department of Integrated Traditional Chinese and Western Medicine, The Second Hospital of Hebei Medical University, Shijiazhuang, Hebei, China; ^4^ Department of Pharmaceutics, School of Pharmaceutical Sciences, Hebei Medical University, Shijiazhuang, Hebei, China

**Keywords:** ultrasound molecular imaging, contrast-enhanced ultrasound, ultrasound contrast agents, preparation, neural network model

## Abstract

There is a need to standardize the process of micro/nanobubble preparation to bring it closer to clinical translation. We explored a neural network-based model to predict the structure-echogenicity relationship for the preparation and fabrication of ultrasound-enhanced contrast agents. Seven formulations were screened, and 109 measurements were obtained. An artificial neural network-multilayer perceptron (ANN-MLP) model was used. The original data were divided into the training and testing groups, which included 73 and 36 groups of data, respectively. The hidden layer was selected from three hidden layers and included bias. The classification graph showed that the predicted values of the training and testing groups were 76.7% and 66.7%, respectively. According to the receiver operating characteristic curve, the accuracy of different imaging effects could achieve a prediction rate of 88.1–96.5%. The percentage graph showed that the data were gradually converging. The predictive analysis curves of different ultrasound effects gradually approached stable value of Gain. Normalized importance predicted contributions for the Pk1, poly-dispersity index (PDI), and intensity account were 100%, 98.5%, and 89.7%, respectively. The application of the ANN-MLP model is feasible and effective for the exploration of the synthesis process of ultrasound contrast agents. 1,2-Distearoyl-sn-glycero-3 phosphoethanolamine-N (methoxy[polyethylene glycol]-2000) (DSPE PEG-2000) correlated highly with the success rate of contrast agent synthesis.

## 1 Introduction

Ultrasonography is a universally available, cost-effective, non-invasive, non-ionizing, real-time, and safe diagnostic modality used in pre-clinical and clinical medicine for diagnosis, molecular imaging, and therapeutics (1, 2). Ultrasound molecular imaging (UMI) is a powerful technique for the study of disease progression, diagnostic imaging, and monitoring therapeutic responses (3, 4). The application potential of UMI has been proven in a large number of pre-clinical studies and in a vast array of disease models (5–7).

With the development of molecular imaging, UMI based on a micro/nanobubble contrast agent is advancing rapidly. Ultrasound contrast agents (UCAs) used in UMI aim to improve tissue resolution, allowing for more scientific diagnosis in diverse imaging studies and clinical applications (8, 9). To improve molecular imaging results, researchers have explored the preparation of different types of targeted ultrasound contrast micro/nanobubbles (10–12). These studies have suggested that to form uniform micro/nanobubbles, diverse lipids and surfactants need to be introduced, and various experimental methods need to be applied (13, 14).

However, the preparation of micro/nanobubbles and the method of targeting ligand attachments are complex, cumbersome, and without uniform standards (15). Furthermore, some techniques used for manufacturing micro/nanobubbles may reduce the yield and stability of the resultant bubbles, resulting in poor imaging enhancement (16). Therefore, there is a need to standardize the process of micro/nanobubble preparation to bring it closer to clinical translation (17).

In this study, to standardize the preparation of microbubble contrast agents, we attempted to build a neural network model to predict the imaging effects of UCAs with different formulations and parameters. This model will be used to guide the production of contrast agents, optimize drug formulations, and select meaningful parameters in the production of UCAs. This model could provide guidance for the future design and application of UCAs.

## 2 Materials and methods

### 2.1 Chemicals and reagents

Lecithin, cholesterol, phosphatidylethanolamine, 1,2-distearoyl-sn-glycero-3-phospho, 1,2-distearoyl-sn-glycero-3-phosphocholine, and 1,2-distearoyl-sn-glycero-3-phosphoethanolamine-N-(methoxy[polyethylene glycol]-2000)(DSPE PEG-2000) were purchased from AVT Pharmaceutical Tech Co., Ltd. (Shanghai, China). Chloroform was obtained from Duksan Pure Chemicals Co., Ltd. (Beijing, China). Methanol was obtained from Thermo Fisher Scientific Co., Ltd. (Shanghai, China). Poly(ethylene glycol)4000 and phosphate-buffered saline (pH 7.4) were purchased from Solarbio Science & Technology Co., Ltd. (Beijing, China). All reagents used in our study were of analytical grade and were used without further purification. 1,2-propanediol was purchased from the Taixing Reagent Factory (Tianjin, China). RPMI 1640 and Dulbecco’s Modified Eagle Medium, were purchased from Thermo Fisher Scientific Co., Ltd.

### 2.2 Preparation of contrast agents

In this study, seven different formulations were screened 98 times and 38 times to obtain the lipid suspension (**Table 1**). Measurements were conducted using a Zetasizer Nano ZS 90 analyzer (Malvern Panalytical Ltd., Malvern, UK) by dynamic light scattering, and a total of 109 measurements were obtained. Size distributions of UCAs and their imaging effects after different production conditions were recorded.

**Table 1:** Seven different formulations for ultrasound contrast agent production.

Formulation	PE	CHOL	DSPE-PEG-2000	DSPC	DSPG	PEG-4000	Solvent
1	+	+	+	–	–	–	1
2	–	–	+	+	+	–	1
3	–	–	–	+	+	+	1
4	–	–	+	+	–	–	2
5	+	+	+	+	–	–	3
6	–	–	+	–	+	–	2
7	–	–	–	+	–	+	2

+, The formulation adds this lipid; -, the formulation does not add this lipid; 1,pure water; 2, phosphate-buffered saline and glycerol; 3, phosphate-bufferedsaline; PE, phosphatidyl ethanolamine; CHOL, cholesterol; DSPE-PEG-2000,1,2-distearoyl-sn-glycero-3-phosphoethanolamine-N-(methoxy[polyethyleneglycol]-2000); DSPC, 1,2-distearoyl-sn-glycero-3-phosphocholine; DSPG, 1,2-distearoylsn-glycero-3-phospho; PEG-4000, poly(ethylene glycol)4000.

The following two fabrication methods were used in our experiment: the film hydration method and the stirring dissolution method. Detailed steps of the two methods are described in the **Supplementary Material**.

All processes were performed in a clean environment and all products were stored at 4°C before use.

### 2.3 Characteristics of lipid bubbles

The morphology of lipid particles was examined using an Inverted Biological Microscope AE2000 (MOTIC, CHINA). Pk1, Pk2, Pk3, intensity, Z-Average, poly-dispersity index (PDI), and Pk1, Pk2, and Pk3 area were measured using the Zetasizer Nano described in Section 2.2 (18). Pk1, Pk2, and Pk3 refer to the average lipid particle size of the main, second, and third peaks. Intensity refers to the average intensity of light scattered by lipid particles. The PDI is a polymer dispersibility index used to describe lipid particle size distribution. Z-Average refers to the average particle size of lipid particles. The Pk1, Pk2, and Pk3 area refers to the area occupied by Pk1, Pk2, and Pk3 peaks, respectively.

### 2.4 Cell culture

The murine mammary cancer cell line (4T1 cells) used in this study was obtained from the Cell Bank of the Type Culture Collection of the Chinese Academy of Sciences (Shanghai, China) and was cultivated in RPMI 1640 medium, 10% fetal bovine serum, 100 IU/mL of penicillin, and 100 mg/mL of streptomycin. The cells were cultured in a 37°C humidified incubator with a 5% carbon dioxide atmosphere. Subculture was performed when cells reached 80–90% confluence. 4T1 cells were grown until logarithmic growth was obtained, and diluted with a medium to create the cell suspension.

### 2.5 Mice tumor model and intervention

#### 2.5.1 Ethics statement

All animal procedures were approved by the Research Ethics Committee of The Second Hospital of Hebei Medical University (2021-AE042).

#### 2.5.2 Animal model

BALB/c mice (female, 4–6 weeks old) were purchased from Beijing Huafukang Biotechnology Company (Beijing, China). A total of 1×10^6^ tumor cells in 30 µL were implanted into the four mammary fat pads on day 0 to establish a breast cancer orthotopic tumor model. Cells were maintained on wet ice during implantation.

The tumor volume (mm^3^) was estimated three times weekly using a digital caliper. The major (D_max_) and minor (D_min_) diameters of each tumor were recorded (mm), and the volume was calculated using the following formula: V_T_= 0.5×D_max_×D_min_^2^ (19).

When the maximum diameter of the tumor was 8-10 mm or the tumor volume was ≥200 mm^3^ (about 8-10 days after the tumor was implanted), the group was evaluated for the effect of the contrast agent. Mice were anesthetized by intraperitoneal injection of 0.1 mL/10 g body weight of 5% chloral hydrate. Then, the mice were fixed on the operating table in a supine position, and a tail vein channel was established for injection.

### 2.6 Ultrasonography

B-mode, D-mode, and contrast-enhanced ultrasound (CEUS) were performed using an X4-12L linear scanner in the frequency range of 4.0–12.0 MHz (VINNO G86, VINNO Technology Co., Ltd., Suzhou, China). First, B-mode imaging of the region of interest (ROI) was performed. The ROI covering the entire liver or tumor was delineated along the border in the B-mode images. Second, D-mode imaging was performed to observe the distribution of blood flow in the ROI. Finally, a CEUS clip was analyzed using internal quantification software CBI (VINNO Technology Co., Ltd.) to determine the peak intensity and area under the curve (AUC). During the above studies, the depth, gain, and other settings did not change (20).

### 2.7 Statistical analysis

Data from the experiments are presented as mean ± standard deviation. An artificial neural network-multilayer perceptron (ANN-MLP) model and receiver operating characteristic (ROC) curve were used. All statistical analyses were done using SPSS 23.0 (IBM Corp., Armonk, NY, USA) and GraphPad Prism 8.0.1 (GraphPad Software, Inc., San Diego, CA, USA) was used to visualize the data. A p-value <0.05 was considered to be statistically significant, and a p-value <0.01 was considered to be highly significant.

## 3 Results

### 3.1 Formulation screening

**Figure 1:**
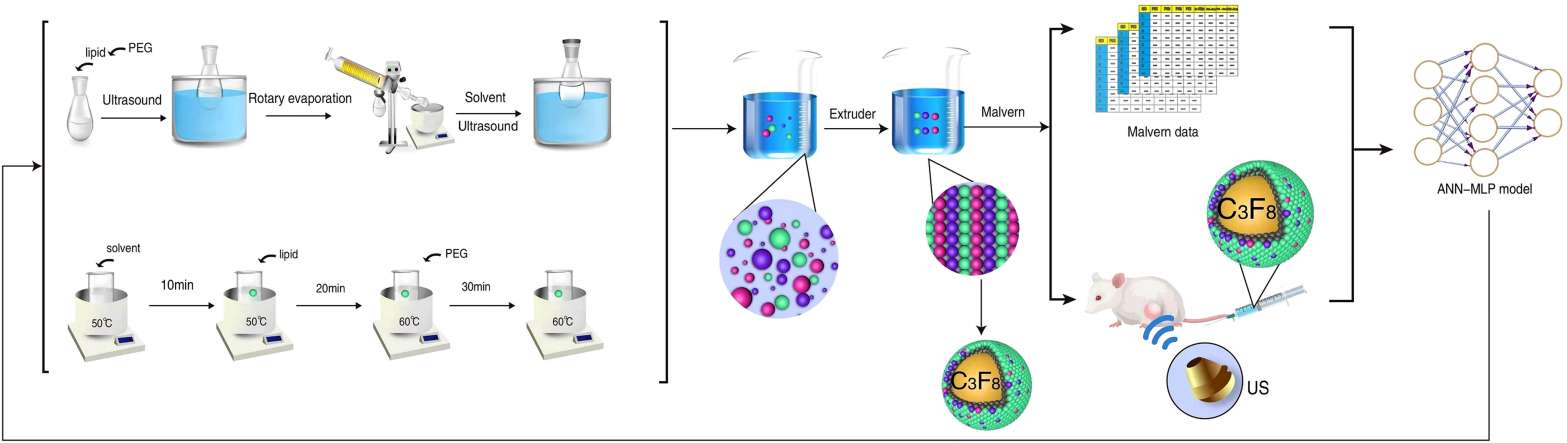
Schematic diagram showing the process of ultrasound contrast agent synthesis and neural network model building.

**Figure 2:**
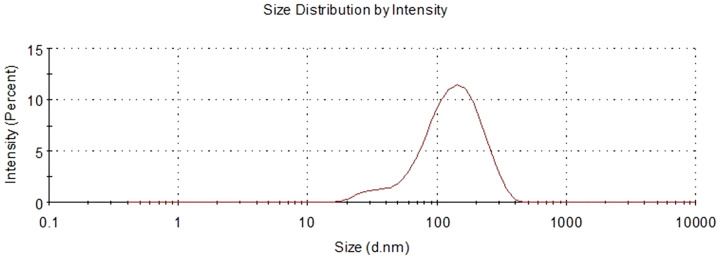
Size distribution of well-imaged UCAs measured by dynamic light scattering.

**Figure 3:**
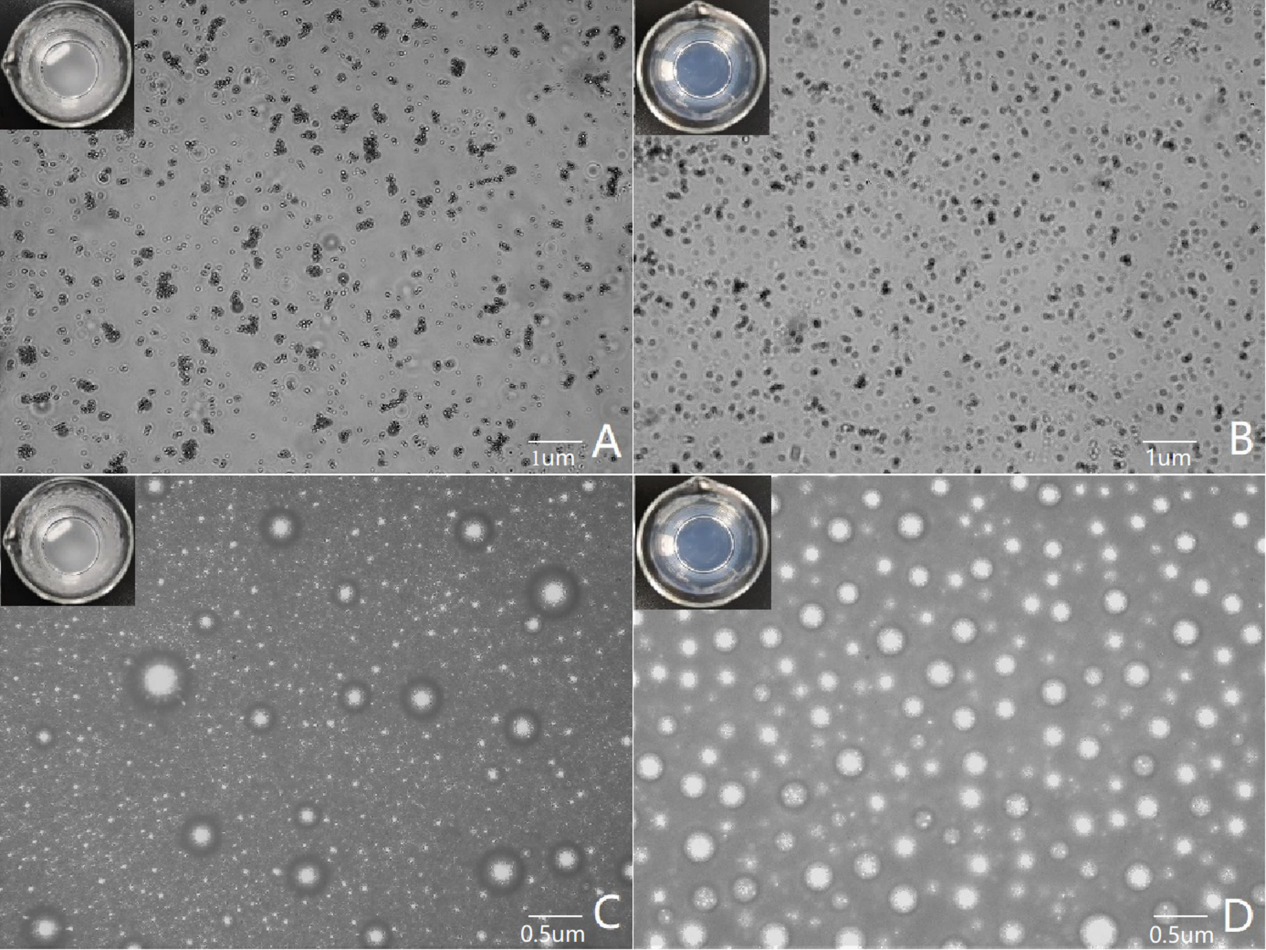
Microscopic images of the contrast of poorly imaged and well-imaged UCAs. **(A, C)** show poor imaging contrast agents, with the lipid suspension in the container appearing cloudy to the naked eye. **(A)** shows that the lipid particles are aggregated under the microscope, and **(C)** shows that the bubbles of the contrast agent are different in size and distributed unevenly after encapsulation. **(B, D)** show good imaging contrast agents, and the lipid suspension in the container is clear and transparent to the naked eye. **(B)** shows that the lipid particles are evenly distributed under the microscope, and **(D)** shows that the bubble size distribution of the contrast agent is uniform after encapsulation.

In total, seven formulations were screened. The experimental flow chart is shown in **Figure 1**. We concluded that formulation 4 was the optimal choice. DSPC and DSPE-PEG-2000 were mixed together in a quality ratio of 5:2 and then dissolved in chloroform and methanol (2:1, vol/vol). The solvent was a mixture of glycerol and PBS (1:9, vol/vol). Size distribution of well-imaged UCAs showed in **Figure 2**. Microscopic images of the contrast of poorly imaged and well-imaged UCAs showed in **Figure 3**.

### 3.2 Data visualization with GraphPad Prism

**Figure 4:**
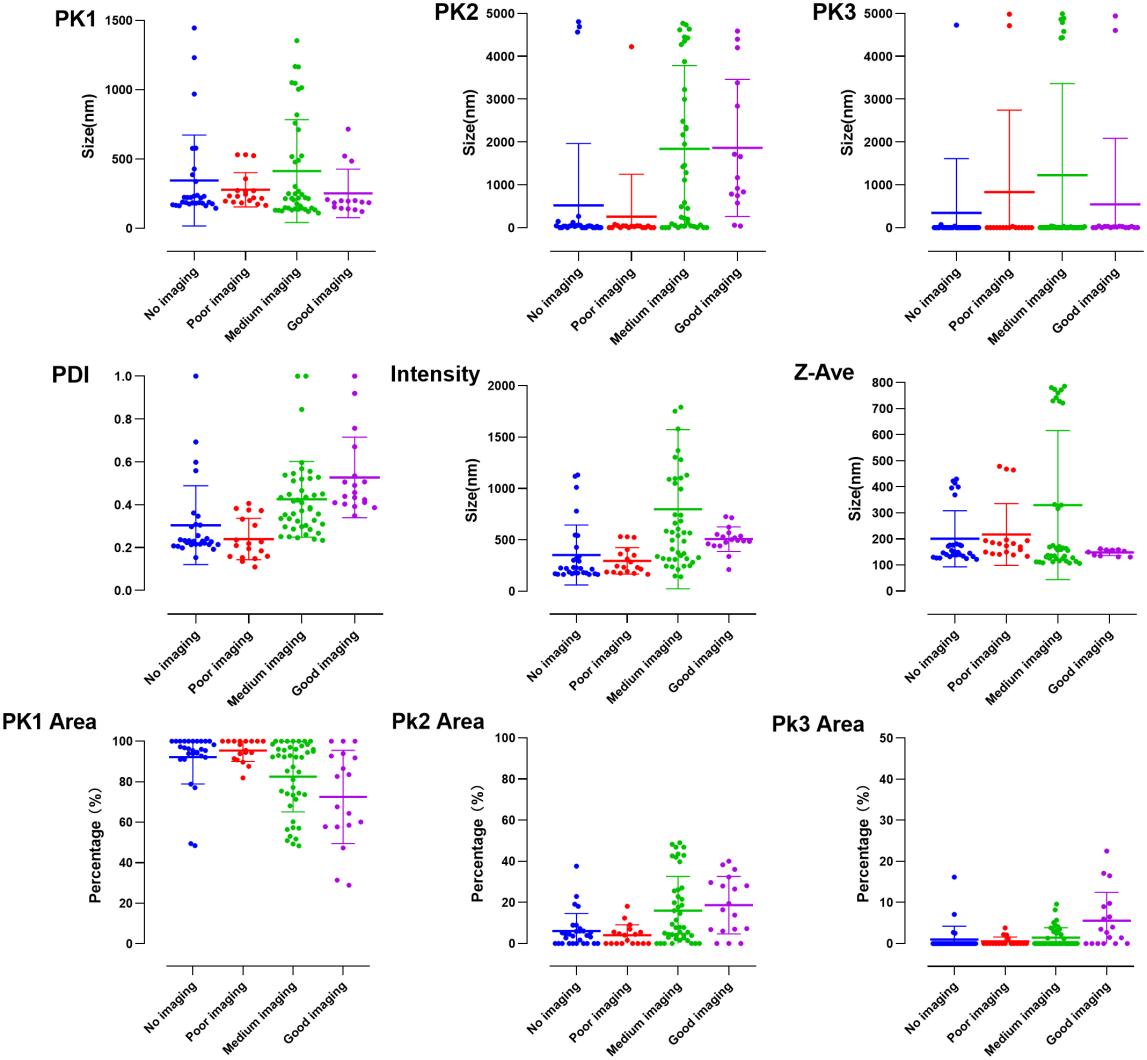
Visualization of particle size data incorporated into the initial conditions of the neural network model.

GraphPad Prism was used to visualize data of the parameters, and values of a good contrast effect were obtained: Pk1, 252.0 ± 175.2 nm; Pk2, 1861.0 ± 1598.0 nm; Pk3, 543.1 ± 1540.0 nm; Pk1 area, 72.5 ± 23.0%; Pk2 area, 18.7 ± 14.0%; Pk3 area, 5.6 ± 6.9%; PDI, 0.53 ± 0.19; intensity, 507.6 ± 118.9 nm; and Z-Ave, 148.5 ± 11.5 nm (**Figure 4**).

### 3.3 The ANN-MLP model

**Figure 5:**
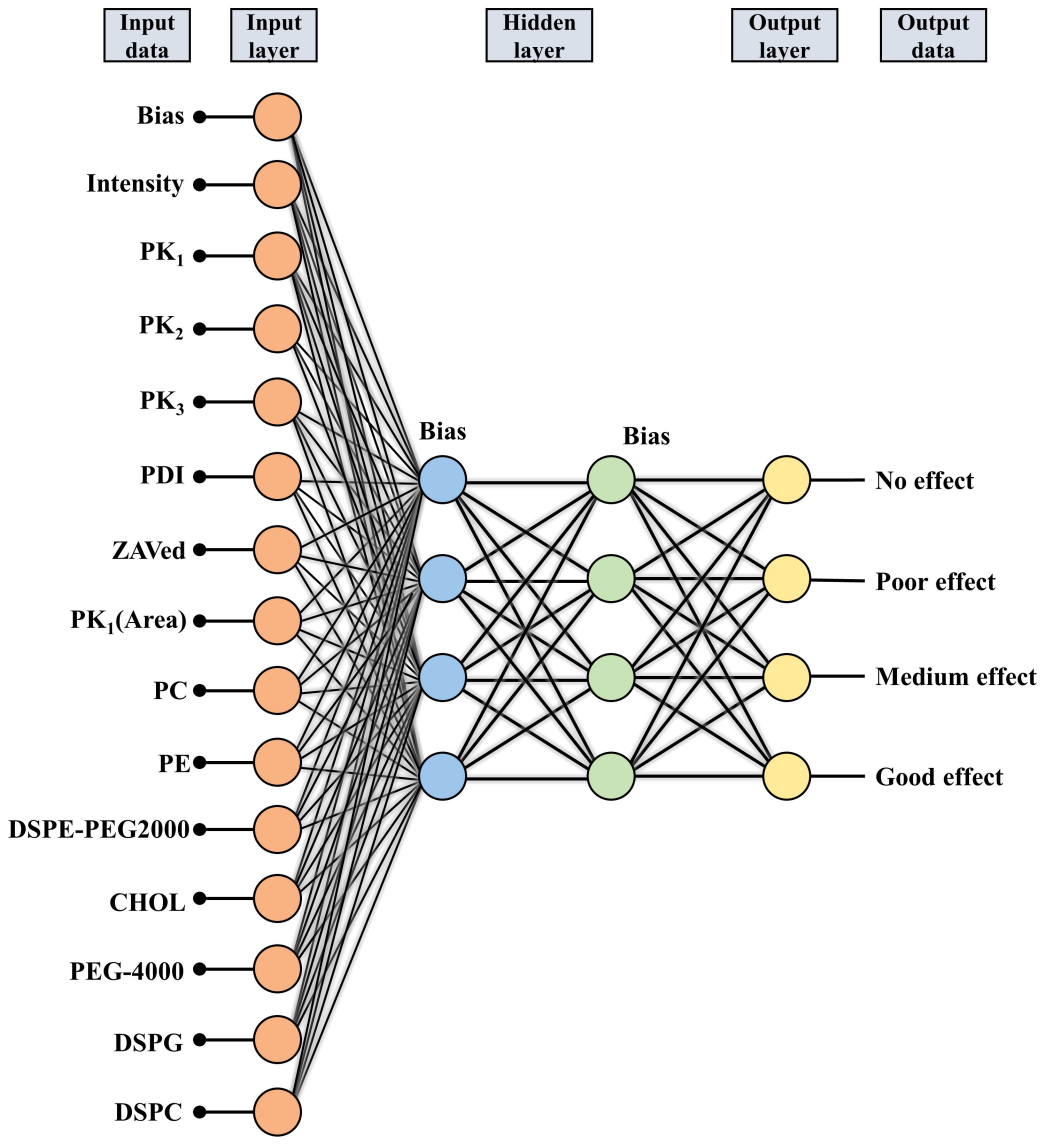
Construction of the neural network used for modeling.

**Figure 6:**
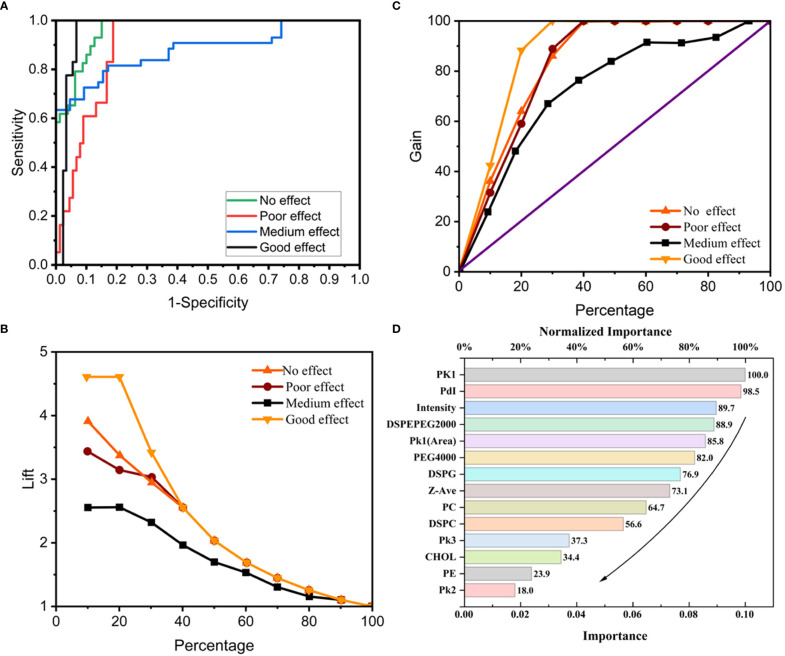
The conclusions of Neural network model prediction and calculation. **(A)** ROC curve of the neural network model; **(B, C)** Percentage graph of the neural network model. **(B)** shows that the data are gradually converging. **(C)** shows that the curves of different ultrasound effects gradually approach stable value of Gain; **(D)** Importance contribution of each index predicted by the neural network model.

**Figure 7:**
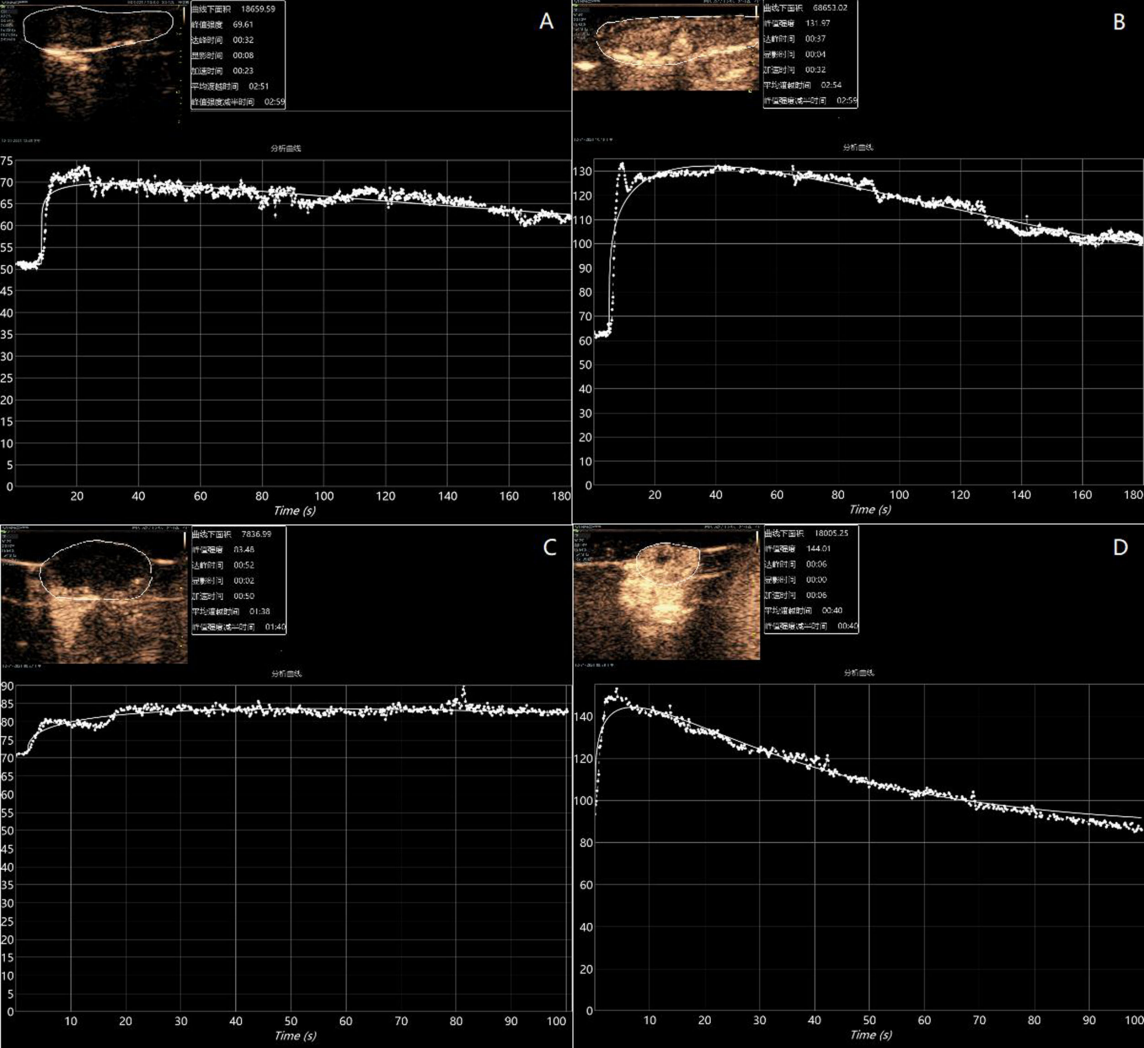
Preparation-based imaging of the liver **(A)** and tumor **(C)** of mice with poorly imaged contrast agents (Pk1: 185.1 nm, PDI: 0.22, and intensity: 181.2 nm). Preparation-based imaging of the liver **(B)** and tumor **(D)** of mice with better imaged contrast agents (PK1: 200.4 nm, PDI: 0.41, and intensity: 496.2 nm).

Analysis of the ANN-MLP model was performed. The original data were divided into training and testing groups, which included 73 and 36 groups of data, respectively. The hidden layer was selected from three hidden layers and included bias. The ANN-MLP neural network model is shown in **Figure 5**. The classification graph showed that the predicted values of the training and testing groups were 76.7% and 66.7%, respectively (**Table 2**). The prediction accuracy of the ROC curve showed that indicators included in different imaging effects could achieve a prediction rate of 88.1–96.5% (**Figure 6A**). The percentage graph showed that the data were gradually converging (**Figure 6B**). The predictive analysis curves of different ultrasound effects gradually approached stable value of Gain (**Figure 6C**). Predicted importance contributions of each index were as follows: Pk1 (the reference standard), 100%; PDI, 98.5%; intensity, 89.7%; DSPE-PEG-2000, 88.9%; and Pk1 area, 85.8% (**Figure 6D** and **Table 3**). We applied the ANN-MLP model to predict the effect of contrast production (**Figures 7A–D**).

**Table 2:** Classification table of the artificial neural network-multilayer perceptron model.

		Predicted percent correct					
Sample contrast effect		No imaging	Poor imaging	Medium imaging	Good imaging	Percent
	No imaging	14	5	0	0	73.70%
	Poor imaging	0	8	4	0	66.70%
	Medium imaging	2	2	26	3	78.80%
Training groups						
	Good imaging	0	0	1	8	88.90%
	Overall percent	21.90%	20.50%	42.50%	15.10%	76.70%
	No imaging	5	5	0	0	50.00%
	Poor imaging	0	4	2	0	66.70%
	Medium imaging	1	1	9	0	81.80%
Testing groups						
	Good imaging	0	0	3	6	66.70%
	Overall percent	16.70%	27.80%	38.90%	16.70%	66.70%

**Table 3:** Normalized importance contribution of each index.

Parameter	Importance	Normalized importance
PC	0.070	64.7%
PE	0.026	23.9%
CHOL	0.037	34.4%
DSPE-PEG-2000	0.096	88.9%
DSPC	0.061	56.6%
DSPG	0.083	76.9%
PEG-4000	0.088	82.0%
ZAved	0.079	73.1%
PDI	0.106	98.5%
Pk1	0.108	100.0%
Pk2	0.019	18.0%
Pk3	0.040	37.3%
Pk1 area	0.092	85.8%
Intensity	0.096	89.7%

PC, lecithin; PE, phosphatidyl ethanolamine; CHOL, cholesterol; DSPE-PEG-2000, 1,2-distearoyl-sn-glycero-3-phosphoethanolamine-N-(methoxy[polyethylene glycol]-2000); DSPC, 1,2-distearoyl-sn-glycero-3-phosphocholine; DSPG, 1,2-distearoylsn-glycero-3-phospho; PEG-4000,poly(ethylene glycol)4000; PDI, poly-dispersity index.

## 4 Discussion

The neural network model has been applied to various imaging examinations including magnetic resonance imaging (MRI), computed tomography (CT) and ultrasound to improve the diagnostic level of images, including disease diagnosis, benign and malignant differentiation (21–23). Neural network models have also been used in the design and optimization of formulation Screening. Cardoso-Daodu IM’s study used an artificial neural network for the optimization of the formulation of liposomes (24). Streba CT’s study applied neural network diagnosis for the screening of contrast-enhanced ultrasonography parameters in the diagnosis of liver tumors (25), allowing for the reduction of experimental efforts significantly toward prescription screening. However, this study sought to address that, currently, there is no application reported for microbubble production.

This study was conducted to address the current inconsistency in contrast agent production standards, mainly in the area of lipid microbubble production. The main purpose was to provide key factors that can be referred to for future lipid contrast agent production. In our study, we made a variety of attempts to explore various methods and appropriate processes, successfully prepare contrast agents, and determine the key parameters and ideal particle size that affect the preparation of contrast agents. Then, we designed an ANN-MLP model of particle structure and synthetic materials for the preparation of UCAs. It was expected that this model would serve as a guide to the stable, controllable, and uniformly sized distribution of UCAs that would provide enhanced contrast for the backscattering signal in ultrasonography.

Our final model showed that the Pk1, PDI, and intensity are the most important parameters affecting the imaging effect of contrast agents. Our model showed that the weight of particle size on the imaging effect was the largest; Pk1 and intensity were the first and third most important factors contributing to the effectiveness. There are many previous studies on the correlation of particle size with the imaging effect of contrast agents. Park et al.’s study showed that particle size has an effect on the contrast effect and that the measured size of the synthetic UCAs between 0.05 and 1 μm is better (26). In our model, Pk1 and intensity were correlated, and the contrast effect was better when Pk1 and intensity were 252.0 ± 175.2 and 507.6 ± 118.9 nm, respectively. The results showed that Pk1 and intensity, which are both nano-level, led to a satisfactory result that may improve the diagnostics value by providing a better contrast effect. First, the main reason for this finding may be that nanoparticles cannot be destroyed by the reticuloendothelial system while maintaining the ability to penetrate the vascular system and accumulate in the tumor tissue *via* passive targeting. Second, nanoparticles can improve poor lymphatic drainage through pores in tumors with diameters ranging from 200 nm to 1.2 µm, which is the structural basis for enhanced permeability and retention effects and enhanced visualization (27).

Moreover, in this model, the PDI was the second most important factor affecting the contrast agent enhancement effect. Previous PDI-related studies, mostly for targeted applications of nanoparticles and microbubbles, described experiments with almost monodisperse particles (polydispersity index <0.2) (28–30). In our model, the enhancement effect was best when the PDI was 0.53 ± 0.19. We can speculate that the contrast-enhancement effect and the drug-carrying effect may be opposite. The particles with the best enhancement effect does not necessarily have the best drug-carrying effect, and conversely, the particles with the best drug-carrying effect have a poor enhancement effect. Thus, we can try to make microbubbles for corresponding purposes by adjusting the value of the PDI, for the better application of contrast agents in clinical settings.

Our model also showed that DSPE-PEG-2000 was highly correlated with the success rate of contrast agent preparation. This result is similar to that of Chen and Borden’s study; the main reason for the impact of DSPE-PEG-2000 on the imaging effect might be that it is coupled with a lipid membrane to ensure the long-term stability of the bubbles and thus decreases interactions with leukocytes or endothelial cells by limiting deposition of protein on the microbubble surface (16, 31, 32).

However, the neural network model we built still has a jitter, and the results of each analysis may be different. This may be because we did not conduct a sufficiently large number of experiments, and the model establishment is not stable, which is also a limitation of this experiment (33). In future research, we should increase the number of experiments to achieve the best and most stable model. When the number of experiments is large enough, the model tends to be stable. However, according to our results, the AUC was >80%, which shows that the establishment of our model is reasonable and meaningful. In addition, the neural network model contains hidden layers, and scientists’ specific understanding of the hidden layer is still limited. All of these limitations require us to increase the sample size and conduct further research in the future.

In future experiments, we will repeatedly train the neural network model. We must keep exploring to determine the various factors that may influence the effect of bubbles and subsequently produce bubbles with a better contrast effect.

## 5 Conclusions

We performed a pre-clinical study of contrast preparation and applied an ANN- MLP model to analyze the results and identify key factors affecting the imaging effect of contrast agents. We applied the ANN-MLP model combined with GraphPad Prism to predict the contrast effects of different contrast agents in mice and verified that our model’s predictions were successful. These key parameters can be used to improve the success rate of contrast preparation in the future.

## Data availability statement

The datasets presented in this study can be found in online repositories. The names of the repository/repositories and accession number(s) can be found in the article/**Supplementary Material**.

## Ethics statement

All animal procedures were approved by the Research Ethics Committee of the Second Hospital of Hebei Medical University (2021-AE042).

## Author contributions

Conceptualization, methodology, design, and writing: FL, BX, WX, YF, WW, HT, SH, and LL. Supervision: YW. All authors contributed to the article and approved the submitted version.

## Acknowledgments

We thank the Pharmaceutics laboratory teachers and students of Hebei Medical University for their help during the experiment. We thank the doctors and students of the ultrasound department of The Second Hospital of Hebei Medical University for their help during the experiment. We also thank Xu Guo (Gambridge Analytica Data Lab) for his contributions in providing methodology of advice on data analysis.

## Conflict of interest

The authors declare that the research was conducted in the absence of any commercial or financial relationships that could be construed as a potential conflict of interest.

## Publisher’s note

All claims expressed in this article are solely those of the authors and do not necessarily represent those of their affiliated organizations, or those of the publisher, the editors and the reviewers. Any product that may be evaluated in this article, or claim that may be made by its manufacturer, is not guaranteed or endorsed by the publisher.
