# Supplementary material for: Preparation of ultrasound contrast agents: The exploration of the structure-echogenicity relationship of contrast agents based on neural network model
Source: Front Oncol. 2022 Oct 5;12:964314. doi: 10.3389/fonc.2022.964314 (PMC9581267; doi:10.3389/fonc.2022.964314)
Supplement: Supplementary file 1 [file DataSheet_1.doc]

Supplementary Material

**The general steps of the film hydration method**

1: Lipid compounds were mixed together and then dissolved in chloroform and methanol (2:1, vol/vol).

2: The above solution was transferred to a round flask for rotary evaporation to form a lipid film.

3: The dried film was hydrated by different solvents for the lipid suspension.

4: Next, the different suspension was repeatedly extruded different times through a 200 nm membrane by mini-extruders (Avanti Polar Lipids, Alabaster, AL, USA).

5: Then, the homogeneous lipid suspension was transferred into a sealed vial, and the air was exhausted and replaced with 99.995% pure perfluoropropane.

6: The mixture was then mechanically vibrated for 60 s in a dental amalgamator (YJT Medical Apparatuses and Instruments, Shanghai, China), and resuspended in 2 ml of sterile vial to activate the bubbles.

**The general steps of stirring and dissolving method:**

Briefly, a beaker was preheated in a 50 °C in a water bath, solvent was added to the beaker, and magnetic stirring beads were added; the lipids were weighed according to different recipes, dissolved according to a 50-60 °C gradient heating method, and stirred thoroughly to obtain a lipid suspension. The particle size was then determined. This solution was then transferred to a 2 mL headspace vial, capped with a rubber stopper, and sealed with an aluminum cap. Following purging of the vial with C3F8 gas, the bubbles were activated by mechanical agitation using a dental amalgamator (YJT Medical Apparatuses and Instruments, Shanghai, China) for 60 s.
